# Necrotizing fasciitis of chest and right abdominal wall caused by acute perforated appendicitis: Case report

**DOI:** 10.1016/j.ijscr.2018.09.036

**Published:** 2018-10-04

**Authors:** Lotfi Rebai, Aziz Daghmouri, Ines Boussaidi

**Affiliations:** Department of Anesthesiology and Critical Care Medicine, Burns and Trauma Center, Ben Arous, Tunisia

**Keywords:** Necrotizing fasciitis, Acute appendicitis, Perforation, Chest, Case report

## Abstract

**Introduction:**

Necrotizing fasciitis is an uncommon infection characterized by a necrotic infection that rapidly diffuse along the fascia and progresses to systemic sepsis. The combined occurrence of necrotizing fasciitis of the chest wall and acute appendicitis is extremely unusual.

**Case presentation:**

A 27-year-old man without any significant medical history presented to the emergency department, in the postoperative course of a laparotomy for perforated acute appendicitis, with septic shock and a large erythematous region over the right abdominal wall. Laboratory evaluation revealed leucocytosis and lactic acidosis. A new surgical exploration revealed a purulent peritonitis with necrotizing fasciitis involving the right lower abdomen, right psoas muscle and right retroperitoneum. On intensive care unit, the patient was managed with intravenous antibiotics and surgical debridement. In the following days, the patient developed extension of the necrosis to the right chest wall. A computed tomography scan of the chest showed right-sided pleural effusion with erosive aspect of the ribs. Necrotic tissues were debrided and antibiotic was changed due to wound superinfection with Acinetobacter Baumannii.

**Discussion:**

Necrotizing fasciitis of chest due to acute appendicitis is extremely unusual. The optimal treatment associates appropriate antibiotics, oxygenation of infected tissue, and surgical debridement.

**Conclusion:**

Acute appendicitis resulting in necrotizing fasciitis of chest is rare but life-threatening. Early diagnosis and treatment is essential to reduce morbidity and mortality.

## Highlights

• Necrotizing fasciitis is an uncommon infection and it remains a life-threatening condition.
• Necrotizing fasciitis of chest caused by acute perforated appendicitis causes a fulminant course and high mortality rate.
• Antibiotic administration, surgery and Hyperbaric oxygen therapy are the appropriate choice for necrotizing fascitis management.

## 1 Introduction

Necrotizing fasciitis (NF) is a bacterial derma-hypodermis affecting the soft tissue and muscular fascia [1]. Its location to the chest wall is rare and it's usually caused by thoracic drainage, lung surgery or esophageal resection [2,3]. The combined occurrence of necrotizing fasciitis of the chest wall and acute appendicitis is extremely unusual and it causes a fulminant course and high mortality rate especially in immunocompromised patients. Early diagnosis and treatment are two main factors responsible for the prognosis. We report the first case of necrotizing fasciitis of the chest wall secondary to a perforated appendicitis. This case has been reported in line with the SCARE criteria [4].

## 2 Case report

**Fig. 1:**
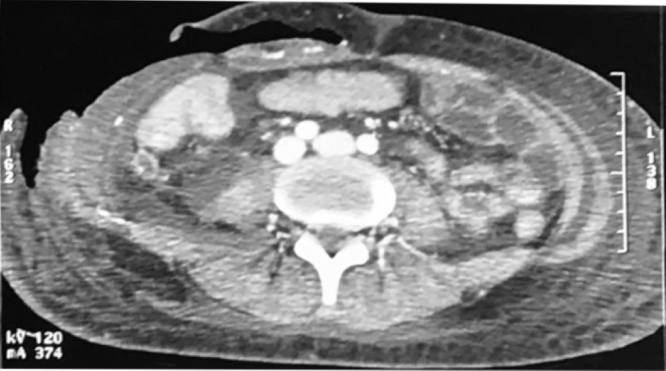
Post-operative axial section of a CT scan shows collections in the right iliac fossa in addition to peritoneal effusion.

A 27-year-old man without any significant medical history, presented to the emergency department (ED) with right lower abdominal pain and a fever of 38.7 °C A computed tomography (CT) scan of abdomen and pelvis showed a perforated appendix (Fig1). Emergency laparotomy was performed and the patient underwent appendectomy and washout. He was discharged home three days later.

On postoperative day 12, the patient was presented again to the ED with a septic shock with a pain of right flank. The vital signs at presentation were as follows: GCS 11/15; blood pressure 110/65 mmHg; pulse 110–120 beats/min; respiratory rate 25 breaths/min and body temperature 39 °C. Physical examination showed right thigh tenderness with a moderately erythematous abdomen and subcutaneous emphysema. Laboratory evaluation revealed a white blood cell count of 25000/mm³ with 90% neutrophil forms; hemoglobin 13 g/dL; Creatinine 65 μmol/L; C-reactive protein was 200 mg/dL and serum lactate level 4.9 mmol/L

A new surgical exploration was performed after appropriate resuscitation with intravenous fluids and antibiotic, revealed a purulent peritonitis with necrotizing fasciitis involving the right lower abdomen, right psoas muscle and right retroperitoneum. Culture of the necrotic tissues revealed polymicrobial infection consisting of *Escherichia coli* and *Pseudomonas aeruginosa*.

**Fig. 2:**
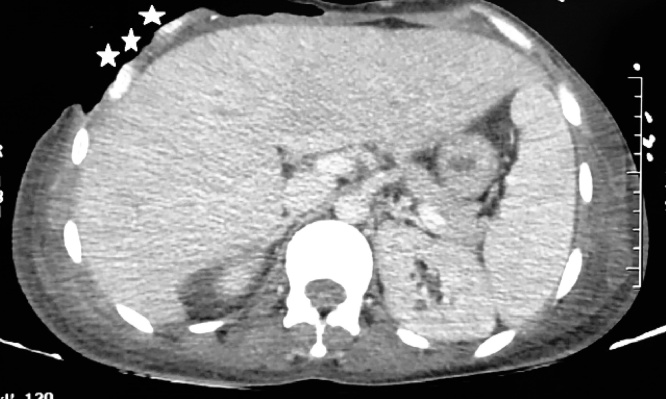
CT scan of the chest showing right-sided pleural effusion with erosive aspect of the ribs (white stars).

**Fig. 3:**
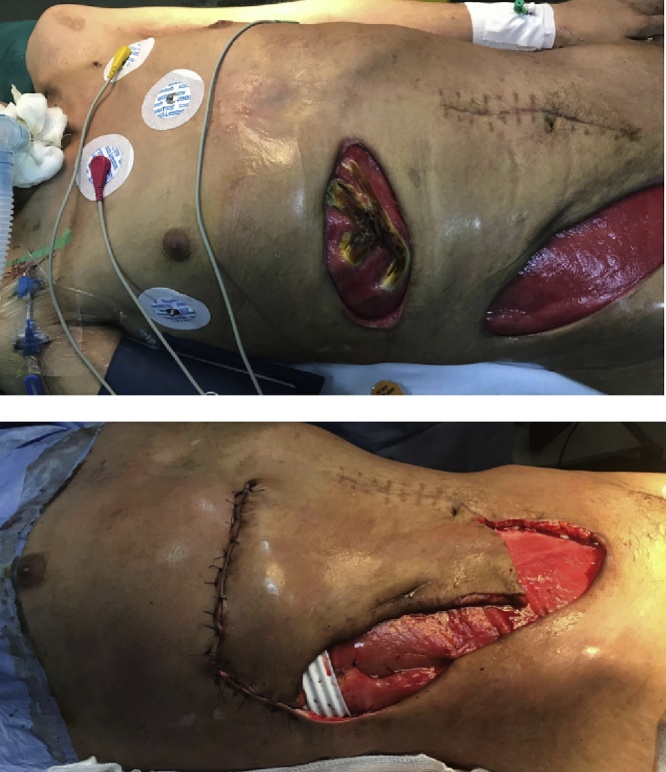
Necrotizing fasciitis of the chest wall before and after the put of the skin flaps by the plastic surgeon.

Postoperatively, the patient stayed intubated in the surgical intensive care unit and had mild hemodynamic instability requiring low-dose of noradrenaline. Antibiotics (Vancomycin 20 mg/kg/24 h, Tazobactam-Piperacillin 80 mg/kg every 6 h, Amikacin 15 mg/kg/24 h, metronidazole 40 mg/kg) were administered and the patient received repeated debridement for the unhealed abdominal wound. In addition, incision, drainage and hyperbaric treatments were performed three times for the necrotizing soft tissue infections over the retroperitoneal region of the right flank and also over the scrotal region and the external genital organ because of the extension of the necrosis. One week later, the patient developed another septic shock due to the extension of the necrosis to the right chest wall. A CT scan of the chest showed right-sided pleural effusion with erosive aspect of the ribs (Fig2). Necrotic tissues were debrided and antibiotic was changed due to wound superinfection with Acinetobacter Baumannii (Coli-mycin 100,000 UI/kg/24 h, rifampicin 30 mg/kg/24 h). After the improvement of his general health, skin flaps were putted by plastic surgeon to cover the exposed ribs (Fig. 3).

On the sixty postoperative day, the patient developed a mitral valve infective endocarditis with negative blood culture. Three days later, he died of septic shock and multiple organ failure.

## 3 Discussion

Necrotizing fasciitis due to a perforated appendix is rarely reported, only 14 cases have been reported in the English literature and the calculated mortality rate was 35% [5]. Anaya et al [6] have demonstrated that infection of the lower extremities is the most common site of NF (57, 8%), followed by the abdomen and the perineum. However, the location on the chest wall is extremely rare. The few reported cases are subsequent to thoracic drainage, lung surgery or esophageal resection [7] but we didn't find any case of necrotizing fasciitis of the chest wall due to a perforated appendix.

Necrotizing fasciitis is a severe, rare, potentially lethal soft tissue infection which progress rapidly, and septic shock may ensue; hence, the mortality rate is high (median mortality 32.2%) [8]. Recent studies have concluded that necrotizing fasciitis can be classified into four types, according to microbiological findings [9]. The most common is type I, also known as the polymicrobial type. Accounting for 70–90% of cases, it typically affects patients with several co-morbidities, such as diabetes mellitus.

There is no age or gender predilection, but higher rates of NF are seen in obese, diabetic, and immunocompromised patients, as well as alcoholics and patients with peripheral vascular disease. However, NF can (and do) occur in young, otherwise healthy patients with none of these predisposing factors [9] and our case is an extremely rare example of the NF in a young patient without any medical history.

Early diagnosis and treatment are two main factors responsible for the prognosis. The symptoms of disease are not characteristic but patients with NF usually present local pain and erythema [10]. As the infection develops, the pain becomes more intense with appearance of symptoms of general toxicity including fever, dehydration, confusion, diarrhea, vomiting and weakness. As a result, the patient displays hypotension, elevated white blood cell count, metabolic acidosis, coagulopathy. In this late stage of the disease, the patient looks apathetic and indifferent. Imaging investigation can help to establish the diagnosis of NF especially in equivocal cases [6,11].

There is no cure without complete excision of the non-viable tissues. The antibiotic treatment must be started immediately, even before the results of the microbiological analyses. Antibiotics are usually adjuvant to the surgical treatment because the local vascular thrombosis results in poor antibiotic tissue diffusion. The recommended antibiotic treatment consists in the association of β-lactamine, imidazole ± aminoside [12]. The mean duration of antibiotic therapy for NF is 4–6 weeks [13].

Lately, many surgeons worldwide have started using vacuum-assisted closure (VAC) therapy for fast and effective wound closure[14]. Several randomized studies have demonstrated improved wound healing and a significant reduction of wound surface area in full-thickness wounds treated with VAC devices because it has several benefits in wound management, with wound area reduction and formation of granulation tissue being the most prominent.

For the Hyperbaric oxygen (HBO) therapy, multiple studies have examined the use of HBO in the treatment of NF with mixed results but with the lack of proven benefit from HBO, and significant limitations on care delivery possible in hyperbaric chambers, we do not recommend routine consideration of HBO therapy for patients with NF and feel its use should only be considered in hemodynamically stable patients in whom HBO therapy will not delay surgical debridement [15,16].

## 4 Conclusion

In conclusion, we have reported a case of necrotizing fasciitis of the chest, abdominal wall and the scrotal region as a complication of a perforated appendicitis which is extremely uncommon especially in a young patient without any past medical history. We think that is one of the first case of thoracic injury caused by a complicated appendicitis reported in literature. Early diagnosis and treatment is essential to reduce morbidity and mortality.

## Conflict of interest

The authors declare no conflicts of interest.

## Funding

No source of funding

## Ethical approval

The ethical committee of the hospital gave the agreement to report this case.

## Consent

Written informed consent was obtained from the patient parents for publication of this case report and accompanying images. A copy of the written consent is available for review by Editor-In-Chief of this journal.

## Author contribution

Study conception and design: REBAI LOTFI, BOUSSAIDI INES

Acquisition of data : REBAI LOTFI, BOUSSAIDI INES, DAGHMOURI AZIZ

Analysis and interpretation of data : REBAI LOTFI, BOUSSAIDI INES, DAGHMOURI AZIZ

Drafting of manuscript: REBAI LOTFI, BOUSSAIDI INES, DAGHMOURI AZIZ

Critical revision: REBAI LOTFI

## Registration of research studies

This is a case report not a research or a study, so Registration of Research Studies is not required.

## Guarantor

Dr. Lotfi Rebai.

## Provenance and peer review

Not commissioned, externally peer-reviewed.
